# Bridging the Heart and Brain—Grand Challenges in the Diagnosis and Management of Cardiovascular and Cerebrovascular Diseases

**DOI:** 10.3390/diagnostics15212726

**Published:** 2025-10-28

**Authors:** Sonu M. M. Bhaskar

**Affiliations:** 1 Global Health Neurology Lab., Sydney, NSW 2150, Australia; sonu.bhaskar@globalhealthneurolab.org; 2 Clinical Sciences Stream, Ingham Institute for Applied Medical Research, Liverpool, NSW 2170, Australia; 3 NSW Brain Clot Bank, NSW Health Pathology, Sydney, NSW 2170, Australia; 4 Department of Neurology & Neurophysiology, Liverpool Hospital, South West Sydney Local Health District, Liverpool, NSW 2170, Australia; 5 Department of Neurology, Division of Cerebrovascular Medicine and Neurology, National Cerebral and Cardiovascular Center (NCVC), Suita 564-8565, Osaka, Japan

Cardiovascular and cerebrovascular diseases (CVDs) remain the leading global causes of mortality and long-term disability [1,2]. Collectively responsible for nearly one-third of all deaths worldwide, they impose a rising economic and societal burden, particularly among aging populations [3] and in low- and middle-income settings [2]. Despite substantial progress in acute intervention, secondary prevention, and imaging, diagnostic and therapeutic gaps persist in early disease detection, risk stratification, and equitable access to advanced care [4,5].

This Special Issue of *Diagnostics*, entitled “*Grand Challenges in the Diagnosis and Management of Cardiovascular and Cerebrovascular Diseases*”, brings together original research and comprehensive reviews that reflect the growing convergence between cardiovascular and cerebrovascular medicine. The eleven papers featured span neurovascular imaging, genetic and inflammatory biomarkers, artificial intelligence-driven prediction models, and early identification frameworks for heart failure. Together, these contributions highlight the need for multidisciplinary collaboration and global standardization in diagnostic practice [6].

Accurate diagnosis remains foundational to effective vascular management [7]. Several studies in this Special Issue demonstrate how advances in imaging continue to redefine both cardiac and cerebrovascular assessment [8]. The NEUROGEN-SVD Study (Jin et al.) analyzed 18,425 participants to examine MRI-visible markers of cerebral small vessel disease (white matter hyperintensities, lacunes, microbleeds) and the APOE ε4 allele in predicting cognitive outcomes [9]. The findings strengthen the concept of an integrated *vascular–neurodegenerative continuum* and support multimodal imaging-genetic models for risk assessment and early detection of cognitive impairment [10]. In coronary imaging, Singhal et al. reported that computed tomography coronary angiography (CTCA) performed at initial presentation significantly improved diagnostic accuracy in children with Kawasaki disease [11]. Conducted at a large tertiary care hospital in North India, the study demonstrated that CTCA identified distal aneurysms and coronary thrombi frequently missed on transthoracic echocardiography, thereby facilitating more precise treatment decisions and, in several cases, allowing for the appropriate de-escalation of therapy. Bonanni et al. evaluated global longitudinal strain (GLS) after surgical aortic valve replacement. GLS improved significantly after one year, particularly among patients with mild diastolic dysfunction, suggesting that strain metrics are sensitive indicators of myocardial remodeling and postoperative recovery beyond conventional ejection fraction [12]. Together, these studies demonstrate the increasing role of quantitative imaging parameters and how modern imaging enhances diagnostic accuracy, treatment planning, and longitudinal assessment in vascular disease.

The contribution of systemic inflammation and circulating biomarkers to vascular disease pathophysiology also features prominently in this Special Issue [13]. Memiş et al. examined the prognostic role of the neutrophil-to-lymphocyte ratio (NLR) in over 1000 patients with large-vessel occlusion stroke undergoing thrombectomy [14]. While NLR correlated with initial stroke severity, it did not independently predict 90-day outcomes after successful reperfusion, suggesting that its prognostic value may be limited to the acute inflammatory phase [15]. On a population scale, Sastre-Alzamora et al. analyzed data from 139,634 Spanish workers and demonstrated that atherogenic indices (total cholesterol/HDL, LDL/HDL, and triglyceride/HDL ratios) strongly correlated with elevated “*Heart Age*” [16]. These accessible and low-cost markers have potential utility in population-level screening and risk communication, bridging laboratory science and preventive cardiology.

Genomic and molecular studies continue to advance precision diagnostics in cardiology [17]. Jiang et al. identified two novel truncating mutations in SOX4 among patients with familial and idiopathic atrial fibrillation (AF) [18]. Functional assays confirmed impaired transactivation of GJA1 (connexin-43) and SCN5A, implicating SOX4 as a contributor to atrial electrical remodeling. These findings expand the molecular understanding of AF and highlight how genetic testing can inform individualized risk profiling and early detection strategies [19,20].

Artificial intelligence (AI) is increasingly being integrated into vascular diagnostics [21]. Goh and colleagues conducted a meta-analysis of machine learning (ML) models for stroke and AF across more than seven million patients [22]. The pooled area under the receiver operating curve (AUROC) ranged between 0.68 and 0.79, indicating moderate accuracy with superior performance observed in ensemble approaches such as Random Forest and Extreme Gradient Boosting. Despite these promising results, the analysis revealed major gaps in validation, calibration, and reporting standards. The study reinforces that AI tools must be transparent, reproducible, and population-representative to achieve reliable clinical translation. Standardized evaluation frameworks, including TRIPOD-AI guidelines, remain essential for benchmarking their real-world utility.

Interventional and critical care aspects of cardiovascular management are also represented. Ziayee et al. compared outcomes in patients treated with veno-arterial extracorporeal membrane oxygenation (va-ECMO) alone versus ECMO combined with intra-aortic balloon pump (IABP) [23]. No significant difference in procedural brain infarction rates was observed between groups, and thromboembolic strokes predominated. These results suggest that IABP use neither increases nor mitigates the neurological risk inherent in ECMO but underscores the importance of integrated neuro-monitoring and anticoagulation management in these high-risk populations.

Two comprehensive reviews in this Special Issue address heart failure (HF), emphasizing early detection and stratified care [24,25]. Jankajova et al. introduce a six-stage HF framework linking behavioral and biological risk factors with advanced imaging parameters, including speckle-tracking echocardiography and global longitudinal strain, to identify subclinical dysfunction before symptom onset [24]. Manzi et al. reviewed acute HF across non-ischemic cardiomyopathies, delineating significant heterogeneity in etiology, clinical presentation, and prognosis [25]. Both studies advocate for substrate-based diagnostic strategies and targeted therapies, key steps toward the realization of precision cardiology.

Beyond individual modalities, this Special Issue highlights an overarching imperative: the integration of diagnostic data into coherent, clinically actionable frameworks. Cardiovascular and cerebrovascular disorders are physiologically interconnected manifestations of systemic vascular dysfunction. Progress, therefore, depends on harmonizing molecular biomarkers, imaging metrics, and computational models to capture the full spectrum of vascular health and disease. The globally diverse studies represented here, spanning Asia, Europe, and Australia, underscore the shared commitment to addressing these challenges collaboratively. Yet, equitable access remains central; diagnostic innovation must be accompanied by implementation strategies that ensure applicability across varied healthcare systems and populations.

This Special Issue of *Diagnostics* highlights current progress and ongoing challenges in the diagnosis and management of cardiovascular and cerebrovascular diseases. Advances in imaging, molecular profiling, genetics, and AI are reshaping clinical practice by improving diagnostic precision and guiding individualized management [17,21]. However, further work is needed to standardize methodologies, validate tools across populations, and embed innovations within clinical workflows or everyday clinical practice. Multidisciplinary collaboration and cross-regional partnerships will be essential to translating technological advances into tangible improvements in patient outcomes. The integration of cardiovascular and cerebrovascular diagnostics represents an important step toward a unified approach to vascular health, one that aligns technological innovation with patient-centered, equitable care.
